# Development and validation of a brief self-assessed wisdom scale

**DOI:** 10.1186/s12877-020-1456-9

**Published:** 2020-02-12

**Authors:** Sai-fu Fung, Esther Oi-wah Chow, Chau-kiu Cheung

**Affiliations:** Department of Social and Behavioural Sciences, City University of Hong Kong, Kowloon Tong, Hong Kong, China

**Keywords:** Wisdom, SAWS, BSAWS, Confirmatory factor analysis, Older adults

## Abstract

**Background:**

This longitudinal study aimed to develop a nine-item Brief Self-Assessed Wisdom Scale (BSAWS) derived from the original 40-item Self-Assessed Wisdom Scale (SAWS).

**Methods:**

The psychometric properties of the shortened scale were evaluated based on a sample of 157 older adults. The factor structure and dimensionality of the original SAWS were examined using confirmatory factor analysis. Subsequent explorative factor analysis of the BSAWS supported the construct validity of the shortened scale.

**Results:**

The internal consistency, convergent validity and construct validity of the shortened scale were also evaluated and the results indicated that the BSAWS possesses good psychometric properties and is comparable with the full version.

**Conclusions:**

This scale refinement may help researchers and practitioners conduct epistemological surveys or clinical research related to wisdom.

## Background

Wisdom is an ancient construct with a long history of conceptualisation based on normative approaches across cultures, ranging from Greek philosophers such as Socrates and Aristotle to Chinese philosophers such as Confucius. In recent years, the concept of wisdom has been further revitalised in empirical research on social and positive psychology [1–4]. The latest empirical research on wisdom can be broadly categorised into two domains [5]. The first one is performance measure of personal wisdom, also known as Berlin wisdom paradigm, involves the analysis of wisdom-related performance in laboratory setting with trained raters to transcribe the responded data introduced in the 1980s [6, 7]. In recent years, this explicit theory based method was utilised and adapted in different contexts, such as in Australia [8], China [9], Germany [10] and United States [11]. The focus of this paper is the second approach, i.e., latent factor analyses of wisdom that mainly rely on using self-reported survey methods to assess wisdom, such as Self-Assessed Wisdom Scale (SAWS) [2], Three-dimensional Wisdom Scale [12], Practical Wisdom Scale and Transcendent Wisdom Scale [13] and Wisdom Development Scale [14], etc. We acknowledge that the above constructs indeed provide important tools for researchers and practitioners to study the issues related to different facets of wisdom. The purpose of this study is not to compare the wisdom constructs, which have been widely discussed and debated by scholars, both normatively and with empirical evidence [3, 15–20]. Rather, we embrace the idea that each wisdom construct has its own merits and the variety of constructs can enhance our understanding of wisdom across different dimensions and situations.

Nevertheless, the abovementioned latent factor analyses of wisdom constructs suffer from two limitations. First, the scale developers did not employ the latest validation tools to evaluate the dimensionality and factor structure of the scales. These scales were developed with a sole reliance on the exploratory factor analysis (EFA) results to identify the factor structure, without verification by a confirmative factor analysis (CFA) [2, 13]. Second, while some studies have attempted to validate the wisdom scales with a CFA, the adopted cut-off criteria were far below the current standards, and the scales also suffer from problems like a lack of internal reliability [2, 5, 12, 21, 22]. To solve the above problems, many wisdom scales have been revised, validated and adapted into different languages or developed into shortened versions for ease of use [12, 17–19, 23–26]. Yet, there is paucity of study examined the dimensionality and validity of SAWS with the latest psychometric tools which warrant our attention.

The Self-Assessed Wisdom Scale (SAWS), a self-reported instrument for measuring wisdom at the individual level, has been widely used by researchers and clinical practitioners. SAWS focuses on five dimensions, namely experience, reminiscence, openness, emotion regulation and humour, and has received positive evaluations of its internal consistency and psychometric properties [2, 18, 19, 23, 27]. Numerous studies have used SAWS to explore the relationships between wisdom and various psychosocial outcomes. JD Webster [28] suggested that wisdom is positively associated with psychosocial characteristics derived from the Erikson tradition, such as ego-integrity, life attitudes and values. Using hierarchical regression analysis, JD Webster, GJ Westerhof and ET Bohlmeijer [29] identified a positive relationship between wisdom and mental health among Dutch adults. The balanced time perspective also uniquely predicted both mental health and wisdom in a sample of 512 adults in the Netherlands [30]. JD Webster and XC Deng [31] used the wisdom scale to study the relationship between traumatic life events and mental health outcomes among 320 respondents in Canada. A later study further suggested that wisdom and meaning contribute to positive self-development in areas such as optimism, self-esteem and self-characteristics in emerging adulthood [27]. In a recent study, C Cheung and EO Chow [32] identified a positive relationship between wisdom and well-being among older Chinese.

Despite the widespread application of SAWS, few studies have managed to fully replicate its original factor structure. Although SAWS possesses good internal consistency and convergent validity [23, 27], its factor structure and dimensionality are inconclusive and subject to a number of limitations [17, 19]. First, to date, no studies have used CFA to validate the 40-item five latent factor structure of the scale. JD Webster [23] used CFA to analyse five sub-scale factors used to predict the latent construct wisdom rather than analysing all of the 40 items. Second, some of the SAWS items have a complicated factor structure. For example, JD Webster [2] reported that the ‘humor and openness dimensions have some overlap and weaker loadings’ (p. 16). In particular, items 12, 27 and 17 share the attributes of emotion regulation and reminiscence, items 14 and 24 are related to both emotion regulation and humour and items 5 and 20 are related to openness and humour.

The cross-cultural differences in wisdom that may also account for studies unable to replicate the factor structure. Controversies have arisen when studies have attempted to adapt the scale to other contexts. P Alves, L Morgado and Bd Oliveira [33] attempted to validate a Portuguese version of the 40-item SAWS, but their EFA results showed that the factor structure was significantly different from that of the original scale. In response, the authors proposed five alternative wisdom domains, namely reflection, mood, emotional self-regulation, experience and open mindedness, which are significantly different from those of the original SAWS. Due to the mixed findings on the factor structure of the wisdom scale, A Urrutia, GM de Espanes, C Ferrari, G Borgna, AM Alderete and F Villar [34] combined the 40-item SAWS and 79-item Wisdom Development Scale (WDS) to obtain a shortened 20-item scale with a three-factor structure for studying wisdom related issues. They applied the shortened scale in a study based on a sample of older adults in Argentina. However, their CFA results suggested very marginal model fit.

Given the controversies surrounding the full version of SAWS, this study explores whether the factor structure and dimensionality of the scale need further refinement. As JD Webster [2], who developed the original scale, stated, ‘continued refinement of specific scale items may eliminate those which explain little overall variance’ (p. 21). The first part of this study shows that the full 40-item scale fails to replicate the factor structure of SAWS using CFA. However, the EFA results support the development of a unidimensional nine-item Brief Self-Assessed Wisdom Scale (BSAWS). In the next section, the psychometric properties of the newly proposed BSAWS are evaluated and various tools are used to examine its internal consistency, convergent validity and construct validity. Overall, the results show that the BSAWS provides an efficient and valid tool for assessing wisdom using empirical data and psychometric evidence in different cultural contexts, i.e., Chinese culture.

## Methods

### Participants

This study used a longitudinal repeated measures design with 157 community-dwelling older adults from older adult service centres in Hong Kong [35–37]. According to Table 1, the respondents were aged 72.8 years on average (SD = 8.55) and participated in the study on a voluntary basis. For the inclusion criteria, the respondents possessed sufficient cognitive ability (with 7.9 years of education on average) to understand and respond to the self-reported questionnaire as well as have capacity to provide consent for participation in this study. The sample comprised 25.5% male and 74.5% female respondents. There were four waves of data collection: the initial study (study 1; *n* = 157) was conducted in June 2016. The respondents then completed the questionnaire again after one (study 2; *n* = 136), two (study 3; *n* = 135) and eight (study 4; *n* = 98) months. The research team strictly adhered to the relevant ethical standards and the project was approved by the university’s research ethics committee.

**Fig. 1:**
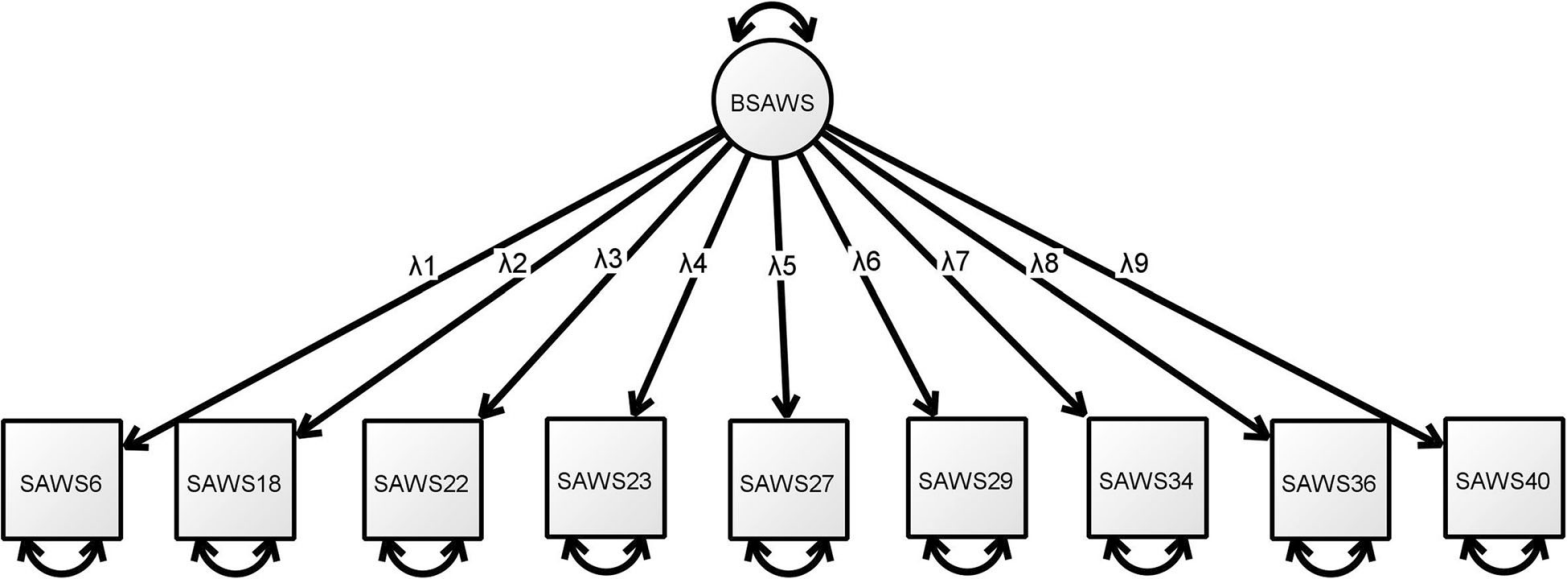
Estimated model of the nine-item BSAWS

**Table 1:** Participant demographic characteristics

Variable	Respondents (n = 157)
Age mean (SD)	72.8 (8.55)
Gender n (%)	
Male	40 (25.5%)
Female	117 (74.5%)
Education level n (%)	
No formal education	26 (16.6%)
Primary education	50 (31.8%)
Secondary education	45 (28.7%)
Tertiary education	30 (19.1%)
Missing	6 (3.8%)
Martial status n (%)	
Single	15 (9.6%)
Married	64 (40.8%)
Divorce/separated	15 (9.6%)
Widowed	62 (39.5%)
Other	1 (0.6%)

### Measurement

The latest SAWS comprises 40 items that measure five dimensions: emotion regulation (items 32, 2, 22, 12, 27, 7, 14, 24 and 17), reminiscence (items 12, 27, 17, 8, 28, 23, 13, 18, 3 and 33), openness (items 35, 25, 30, 38, 5, 20 and 34), experience (items 26, 6, 16, 21 and 1) and humour (items 14, 24, 5, 20, 39, 19, 29, 4, 9 and 10). The respondents were asked to indicate their level of agreement on a Likert-type scale ranging from 1 = *strongly disagree* to 6 = *strongly agree* [2, 18, 23].

### Procedure

The interviewers administered the questionnaire to the respondents at 13 older adult service centres located in different districts in Hong Kong. The items were translated into Chinese using the back-translation procedure [38]. The research team was recruited translator to translate the scale from English to Chinese and then back translated from Chinese to English by the other translator. The back translated version has been verified by the original SAW scale developer JD Webster to confirmed its semantic and conceptual equivalency and avoided any potential cross-cultural biases [39, 40].

CFA was used to replicate and evaluate the construct validity of the SAWS and BSAWS [41–43]. The CFA estimator used diagonally weighted least squares (DWLS) due to the ordinal nature of the Likert scale. DWLS is regarded as less biased and a more optimal fit for this type of scale [44–49]. The results for the following criteria indicated adequate model fit: CFI > 0.95, TLI > 0.95, RMSEA < 0.08, SRMR < 0.08 [41, 50–52]. In addition to these measures, χ2 / df ≤ 3 can be used to determine acceptable model fit [53–56].

Factor analysis with the principal component estimation method was used to evaluate the dimensionality and factor structure of the BSAWS [2, 33, 50, 57]. The Kaiser-Meyer-Olkin (KMO) and Bartlett’s tests of sphericity were used to evaluate the model. The KMO estimates were over 0.70 and the Bartlett’s test was significant (*p* < 0.01), thus confirming that the model had a satisfactory factor structure [58].

In addition, various psychometric testing tools and validated instruments were used to examine the newly proposed BSAWS. The internal consistency of the scale was assessed using Cronbach’s alpha [59] and by examining the corrected item-total correlation between the nine items [50, 60].

The convergent validity was evaluated using other validation constructs reported in the literature on latent factor analyses of wisdom. The wisdom construct was reported to be significantly positively correlated with well-being, self-esteem and other wisdom measures [5, 61–65]. Hence, this study used the following well-established scales to evaluate the convergent validity of the BSAWS: the Personal Well-being Index (PWI) [66, 67], Rosenberg self-esteem (RSE) scale [68–73] and dimensions of the WDS [5, 21]. Research also suggests that wisdom is negatively correlated with depression symptoms [2, 74, 75]. Hence, we used the Geriatric Depression Scale (GDS) [76–78] to evaluate the relationship between depression and the two wisdom scales. The above analysis was implemented using IBM SPSS 25.0 and the R (3.6.0) computing software with lavaan package 0.6–3 [79].

## Results

Table 2 shows the CFA results for the original SAWS and variations of the factor structure in the literature [2, 23, 33]. The CFA results based on study 1 (*n* = 157) suggested that the original full version of SAWS (Model 1) failed to fit the model, with χ^2^ (1570.703) / 510 = 3.08, *p* < 0.001, SRMR = 0.121, CFI = 0.887, TLI = 0.876 and RMSEA = 0.126. Similarly, Model 2 failed to fulfil the cut-off criteria for good model fit, as χ^2^ (2135.089) / 692 = 3.09, *p* < 0.001, SRMR = 0.119, CFI = 0.885, TLI = 0.877 and RMSEA = 0.126.

**Table 2:** Confirmatory factor analysis of SAWS and BSAWS

Model	χ^2^	Df	χ^2^/df	RMSEA [90% CI]	CFI	TLI	SRMR
1. Webster et al. (2007)	1570.703	510	3.08	0.126 [0.119–0.133]	0.887	0.876	0.121
2. Alves et al. (2014)	2135.089	692	3.09	0.126 [0.120–0.132]	0.885	0.877	0.119

With reference to the literature on SAWS [2, 23, 33] and the EFA results in Table 3, this study proposes a nine-item BSAWS with a single factor structure (Additional file 1: Appendix). The BSAWS includes the following domains used in the full scale: emotion regulation (items 22 and 27), reminiscence (items 18, 23 and 40), openness (item 34), experience (items 6 and 36) and humour (item 29). The newly proposed BSAWS scores in studies 1, 2, 3 and 4 are 35.529 (SD = 9.14), 36.610 (SD = 8.44), 37.704 (SD = 7.66) and 37.780 (SD = 9.04), respectively.

**Table 3:** Descriptive statistics and factor loadings from the exploratory factor analysis of BSAWS

Item	x̅	SD	sk	ku	*r*_it_	α_iid_	λ
6	4.222	1.742	−0.805	−0.635	0.368	0.807	0.477
18	3.898	1.630	−0.572	−0.752	0.511	0.787	0.636
22	4.331	1.571	−0.905	−0.143	0.582	0.779	0.707
23	3.936	1.636	−0.618	−0.779	0.349	0.808	0.447
27	3.828	1.594	−0.513	−0.728	0.505	0.788	0.643
29	3.790	1.664	−0.447	−0.976	0.579	0.778	0.711
34	3.955	1.499	−0.663	−0.404	0.619	0.775	0.738
36	4.376	1.439	−1.194	0.698	0.534	0.785	0.654
40	3.191	1.769	0.086	−1.365	0.504	0.788	0.646

x̅ mean, *SD* standard deviation, *sk* skewness, *ku* kurtosis, *r*_*it*_ corrected item-total correlations, α_*iid*_ Cronbach’s alpha, if item deleted; λ factor loadings

### Internal consistency and factorial validity

Table 3 presents the descriptive statistics, including the mean, standardised deviation, skewness, kurtosis, corrected item-total correlations and Cronbach’s alpha, if item deleted, for all nine items of the BSAWS based on the data from study 1. The results show that the BSAWS demonstrates good internal consistency. The corrected item-to-total correlations for the BSAWS ranges from 0.349 to 0.619 and Cronbach’s alpha is above the acceptable range, i.e., 0.808. The BSAWS is also significantly positively correlated (*r* = 0.912, *p* < 0.001) with SAWS.

The results of the KMO and Bartlett’s tests of sphericity for the nine-item BSAWS were 0.823 (χ^2^ = 374.389, *p* < .001), thus indicating appropriate scale construction. The EFA results showed that the factor loadings ranged from 0.477 to 0.738 and explained 40.453% of the total variance (Table 3).

### Convergent and concurrent validity

The results from study 1 show the relationships between BSAWS and SAWS and the other construct-related scales suggested in the wisdom literature. Well-being as measured by the PWI has significant moderate positive relationships with SAWS (*r* = 0.363, *p* < 0.001) and BSAWS (*r* = 0.347, *p* < 0.001). The self-esteem scale also possesses a moderate positive relationship with the two scales. SAW and BSAWS are strongly correlated with the WDS, with *r* = 0.730 (*p* < 0.001) and *r* = 0.741 (*p* < 0.001), respectively. The results also show a weak negative correlation between the scales and GDS, with *r* = − 0.290 (*p* < 0.001) for SAWS and *r* = − 0.345 (*p* < 0.001) for BSAWS. The above findings have been replicated in the subsequent studies 2, 3 and 4 (Table 4). To sum up, the nine-item BSAWS is comparable with the full scale and possesses good convergent validity based on the results of Pearson’s correlation coefficient.

**Table 4:** Correlations between SAWS and BSAWS in relation to other construct-related scales

Scale	SAWS	BSAWS	SAWS	BSAWS	SAWS	BSAWS	SAWS	BSAWS
	Study 1		Study 2		Study 3		Study 4	
PWI	0.363***	0.347***	0.469***	0.389***	0.617***	0.584***	0.530***	0.531***
RSE	0.340***	0.357***	0.392***	0.410***	0.329***	0.280***	0.441***	0.488***
WDS	0.730***	0.741***	0.858***	0.783***	0.833***	0.803***	0.798***	0.818***
GDS	−0.290***	−0.345***	−0.258**	−0.240**	− 0.294***	−0.294***	− 0.184*	−0.214*

*PWI* personal well-being index, *RSE* Rosenberg self-esteem, *WDS* wisdom development scale, *GDS* geriatric depression scale

* *p* < 0.05. ** *p* < 0.01. *** *p* < 0.001

### Content validity

To further validate the content validity of BSAWS, CFA was implemented on the data collected from studies 2, 3 and 4. The CFA results for BSAWS (Table 5 and Fig. 1) indicate good model fit, particularly the combined results across studies 2, 3 and 4, with χ^2^ (51.278) / 27 = 1.90, SRMR = 0.040, CFI = 0.996, TLI = 0.995 and RMSEA = 0.049. Overall, the results indicate that the nine-item BSAWS has generally good fit for a unidimensional factor structure without any post hoc modifications.

**Table 5:** Factor loadings and fit indices from the confirmatory factor analysis of BSAWS, by study (see Fig. 1 for the estimated model)

		Study			
Factor/question		2	3	4	Combo
6. I have made important decisions throughout my life.	λ_1_	0.459	0.641	0.710	0.585
18. Reviewing my past gives me a good perspective on my current concerns.	λ_2_	0.775	0.749	0.692	0.732
22. I can easily express my emotions without feeling like I am losing control of the situation.	λ_3_	0.805	0.727	0.760	0.760
23. I often recall the past to see if I have changed since then.	λ_4_	0.506	0.528	0.576	0.538
27. I am good at identifying subtle emotions in myself.	λ_5_	0.668	0.678	0.830	0.721
29. I often use humour to put other people at ease.	λ_6_	0.622	0.582	0.849	0.680
34. Now I know I can truly appreciate the little things in life.	λ_7_	0.818	0.801	0.839	0.815
36. I have learned valuable life lessons with others.	λ_8_	0.643	0.699	0.789	0.705
40. I often wonder about the mysteries of life and what lies beyond death.	λ_9_	0.592	0.554	0.668	0.587
Model fit					
*N*		136	135	98	369
RMSEA		0.055	0.069	0.084	0.049
RMSEA 90% confidence interval		0.000–0.093	0.029–0.105	0.038–0.126	0.028–0.070
SRMR		0.059	0.057	0.064	0.040
χ^2^ (df = 27)		37.984	44.441	45.609	51.278
χ^2^/df		1.41	1.65	1.69	1.90
CFI		0.995	0.992	0.994	0.996
TLI		0.993	0.989	0.993	0.995

*RMSEA* root mean square error of approximation, *SRMR* standardised root mean residual, *CFI* comparative fit index, *TLI* Tucker Lewis index, *Study 2* initial study plus 1 month, *Study 3* initial study plus 2 months, *Study 4* initial study plus 8 months, *Combo* combined across the three studies

## Discussion

The proposed BSAWS possesses good psychometric properties and is comparable with its full-scale version. According to JD Webster, M Taylor and G Bates [19], ‘the SAWS subscales [are] based upon input by a panel of wisdom experts’ (p. 256). The results of this study show that the BSAWS supports the original five domains of wisdom advocated in the original SAWS, i.e., emotion, regulation, reminiscence, openness, experience and humour. Cronbach’s alpha for the BSAWS is 0.808, which is similar to the values ranging from 0.78 to 0.90 reported in the original SAWS studies [2, 23]. The nine-item shortened version of SAWS also possesses good convergent validity. The results show that SAWS and the BSAWS both hold identical correlational direction and magnitude with the other well-established measurements of well-being, self-esteem and depression. Both scales also have very strong and significant positive correlations (*r* = 0.912, *p* < 0.001). The independent-sample t-test results show that no significant differences were observed in both scale scores on sex of the respondents. There were only weak significant correlation between the educational level (*r* = 0.294, *p* < 0.001; *r* = − 0.328, *p* < 0.001) and age (*r* = 0.292, *p* < 0.001; *r* = − 0.265, *p* < 0.001) of the respondents in BSAWS and SAWS scores, respectively. These findings are aligned with the other wisdom constructs that focused on older adults [12].

This study contributes to the measurement of wisdom in the following ways. First, the shortened version of SAWS can help resolve disputes related to the complicated factor structure and dimensionality of the full version of SAWS. The original scale developer and the subsequent validation studies have generally failed to fully replicate the five latent factor structure of the 40 item scale [2, 18, 23, 33, 34]. For example, a recent study showed that some SAWS items did not load on any factor and that the openness dimension had a questionable Cronbach’s alpha of 0.68 [18, 43, 80]. Consequently, some studies have attempted to shorten the scale by forcefully combining SAWS with other wisdom related constructs without using strict validation procedures to examine the psychometric properties of the revised scale [32, 34]. A validated abbreviated version of SAWS can serve as a useful instrument for designing future studies related to wisdom among older adults and other populations.

This study also provides empirical evidence to support the factor structure of the BSAWS using CFA. Numerous SAWS related studies have used only EFA to evaluate the factorial validity of the scale, without verifying the construct validity with CFA [2, 18, 33]. The only SAWS validation study to use CFA was based on five sub-scales, which served as the latent factors for estimating the loadings on the wisdom construct rather than evaluating all 40 items. The results failed to meet the criteria for adequate model fit, with CFI = 0.947 and RMSEA = 0.107 [23]. The CFA results of Models 1 and 2 (Table 2) in this study managed to replicate the problem of analysing the 40 items using a five latent factor structure. The results showed that none of the models were considered to have a good fit. However, the CFA results for the newly proposed nine-item BSAWS fulfilled all of the stringent criteria for determining good model fit in the structural equation modelling literature [50, 51, 55].

The procedure for developing the BSAWS strictly adhered to the recognised scale development and validation principles to avoid the potential problem of overfitting [43, 81]. The sample from study 1 (*n* = 156) was used to conduct EFA to identify the factor structure of BSAWS. The study 2 (*n* = 136), 3 (*n* = 135) and 4 (*n* = 98) samples were then used to verify the scale’s content validity using CFA. In addition, various psychometric evaluation tools were used to examine the internal consistency and convergent validity of the nine-item BSAWS. In short, the BSAWS was found to possess excellent psychometric properties.

This study has the following potential limitations. First, the small sample size may limit the reliability of the results. This limitation may account for why the CFA results in study 4 (n = 98) only yielded a marginally adequate RMSEA value. Some scholars suggested that a minimum sample size (*N* = 100) is preferred for CFA, but simulation studies suggested that the model (*N* > 50) with more items (> 6) per factor may overcome this limitation [82–84]. The research team had difficulty recruiting significant numbers of respondents from the older adult service centres in Hong Kong. However, the longitudinal repeated measures design used in this study may have compensated for this limitation. The second potential limitation is related to the demographic background of the respondents. Specifically, the results based on Chinese older adults in Hong Kong may have limited generalisability. Lastly, the standalone 9-item scale may need further validation, as the participants may potentially affected by responding to the other 31 items in the scale. Thus, further research is needed to replicate our findings or apply the BSAWS in different cultural contexts to verify this refinement of SAWS. With the abbreviated version of SAWS, it enables researchers to further examine the queries related to wisdom and other psychosocial outcomes, such as its relationship with hedonic and eudaimonia well-being [10, 85–87] in future research projects.

## Conclusions

This study developed and validated an abbreviated nine-item version of SAWS. The results suggest that the BSAWS possesses good internal consistency, acceptable model fit for a CFA and is comparable with its 40-item full version. The newly developed scale can provide an efficient and valid assessment of wisdom for older adults. This abbreviated standardised wisdom measure may encourage researchers and practitioners to conduct epidemiological surveys to evaluate the effectiveness of interventions in a clinical setting. However, future work is needed to confirm the psychometric properties of the scale in larger or more generalisable samples.

## Supplementary information

**Additional file 1: Appendix.** Factor structure and dimensionality of SAWS and BSAWS.

## Data Availability

The datasets used and analysed during the current study are available from Esther Oi-wah Chow on reasonable request.

## Abbreviations

BSAWS: Brief self-assessed wisdom scale
CFI: Comparative fit index
ER: Emotion regulation
EXP: Experience
GDS: Geriatric depression scale
HU: Humour
OP: Openness
PWI: Personal wellbeing index
RE: Reminiscence
RMSEA: Root mean square error of approximation
RSE: Rosenberg self-esteem
SAWS: Self-assessed wisdom scale
SRMR: Standardized root mean residual
TLI: Tucker Lewis Index
WDS: Dimensions of the wisdom development scale
