# Supplementary material for: Development and validation of a brief self-assessed wisdom scale
Source: BMC Geriatr. 2020 Feb 12;20:54. doi: 10.1186/s12877-020-1456-9 (PMC7017457; doi:10.1186/s12877-020-1456-9)
Supplement: Supplementary file 1 — Additional file 1: Appendix. Factor structure and dimensionality of SAWS and BSAWS. [file 12877_2020_1456_MOESM1_ESM.docx]

**Appendix.** Factor structure and dimensionality of SAWS and BSAWS

|  | **Item** | **Webster et al. (2007)** | **Alves et al. (2014)** | **BSAWS** |
| --- | --- | --- | --- | --- |
| 1 | During my life I have already overcome many painful facts. | EXP | EXP | - |
| 2 | I easily adjust my emotions to the present situation. | ER | ESR | - |
| 3 | I often relate past with present situations. | RE | REF | - |
| 4 | I am able to laugh in embarrassment situations. | HU | MO | - |
| 5 | I love to read books that challenge me to think differently about many issues. | OP, HU | EXP | - |
| 6 | I have taken important decisions throughout my life. | EXP | EXP | EXP |
| 7 | When I take personal decisions I do not let myself take over by emotions. | ER | ESR | - |
| 8 | I often think about my own past. | RE | REF | - |
| 9 | There may be funny elements even in very difficult life’s situations. | HU | MO | - |
| 10 | Besides my favourite kind of music I like to listen to other musical styles. | HU | MO | - |
| 11 | Throughout my life I have dealt with many types of people. | - | EXP | - |
| 12 | I am in tune with my own emotions. | ER, RE | ESR | - |
| 13 | I often dedicate myself to the remembering of past events. | RE | REF | - |
| 14 | When I face major life transitions I try and find a funny side. | ER, HU | MO | - |
| 15 | I love trying a variety of different ethnic foods. | - | OM | - |
| 16 | I have gone through various moral dilemmas. | EXP | OM | - |
| 17 | I am very good at interpreting my emotional states. | ER, RE | OM | - |
| 18 | Reviewing my past helps me to have a good perspective of my current concerns. | RE | REF | RE |
| 19 | I laugh easily. | HU | MO | - |
| 20 | I often try new things. | OP, HU | OM | - |
| 21 | I have met a lot of the negative side of life (e.g., dishonesty, hypocrisy...) | EXP | EXP | - |
| 22 | I can easily express my emotions without feeling like I am losing control of the situation. | ER | OM | ER |
| 23 | I often recall the past to see if I have changed since then. | RE | REF | RE |
| 24 | At this point in my life it is easy for me to laugh at my mistakes. | ER, HU | MO | - |
| 25 | Controversial works of art play an important and valuable role in society. | OP | OM | - |
| 26 | I went through many difficult changes throughout life. | EXP | EXP | - |
| 27 | I am good at identifying subtle emotions in myself. | ER, RE | OM | ER |
| 28 | Remembering my past helps me understand the important things in my life. | RE | REF | - |
| 29 | I often use humour to put other people at ease. | HU | MO | HU |
| 30 | I like being surrounded by people whose views are quite different from mine. | OP | MO | - |
| 31 | I discovered by myself that "not everything that shines is gold." | - | EXP | - |
| 32 | I am able to control my emotions when the situation demands it. | ER | ESR | - |
| 33 | I often notice that my past can be an important source of knowledge. | RE | REF | - |
| 34 | Now I know I can truly appreciate the little things in life. | OP | ESR | OP |
| 35 | I have a lot of curiosity to know more about other religions and other philosophies. | OP | ESR | - |
| 36 | I have learnt valuable life lessons with others. | - | EXP | EXP |
| 37 | It seems I have a certain gift to understand the emotions of others. | - | OM | - |
| 38 | Reliving past events increases my confidence to live today. | OP | REF | - |
| 39 | To comfort others I often make fool of me. | HU | MO | - |
| 40 | I wonder many times about the mysteries of life and what lies beyond death. | - | REF | RE |

Note: ER: Emotion regulation; RE: Reminiscence; OP: Openness; EXP: Experience; HU: Humour (Webster et al. 2007); ESR: Emotional self-regulation; REF: Reflection; OM: Open-mindedness; EXP: Experience; MO: Mood (Alves et al. 2014)
